# Editorials

**Published:** 1908-10

**Authors:** 


### EDITORIALS

The Business Office of the JOURNAL-RECORD is 1 1-2 to 5 1-2 South Broad St.
The Editorial Office is 1014-15 Century Bldg.
Address all Business Communications to Journal-Record of Medicine, 1 1-2 to 5 1-2 South Broad Street.
Make remittances payable to THE JOURNAL-RECORD OF MEDICINE.
On matters pertaining to the Editorial and Original communications, address Edgar G. Ballenger, M. D., Atlanta, Ga.
Reprints of original articles will be furnished at cost price. Requests for the same should always be made in THE MANUSCRIPT.
We will present, postpaid, on request, to each contributor of an original article, twenty (20) marked copies of THE JOURNAL-RECORD OF MEDICINE containing such article.

### MEDICAL EDUCATION IN GRÉAT BRITIAN AND IRELAND.

The British Medical Journal publishes annually a special "Educational Number," setting forth the regulations guiding the various schools of medicine and dealing generally with the position of medicine in the British islands.

These, as a matter of comparison, should be interesting reading to the profession in other countries.

"The practice of medicine in the United Kingdom, as in all other civilized countries, is regulated by the State, but in the case of Great Britain and Ireland the State has not proceeded by the direct road followed in the German empire, where every aspirant must pass an examination held by the State, the *Staats Examen,* before he has the legal right to practice. The Medical Act of 1858 called into existence the General Council of Medical Education and Registration of the United Kingdom, and entrusted to it the duty of keeping a *Medical Register,* laying it down that the words "legally qualified medical practitioner," "duly qualified medical practitioner," or any words importing a person recognized by law as a medical practitioner or member of the medical profession, when used in any Act of Parliament, shall be construed to mean a person registered under the Act. A valid medical certificate required by any Act of Parliament can be given only by a registered person. Further, only persons so registered can recover in a court of law charges for medical or surgical advice, attendance or operation.'

"The Council has power to require any body granting a qualification for registration to furnish it with full information as to the course of study and examinations through which students must pass before obtaining the qualification in question."

"Official statistics published recently under the authority of the General Medical Council show that the mean length of the curriculum in the case of 1,111 students whose cases were investigated was three weeks less than seven years; only 14 per cent. succeeded in obtaining a qualification in the minimum period of five years, 33 per cent. obtained it in the sixth year, 18 per cent. in the seventh year, and 13 per cent. in the eighth year. When the remaining 20 per cent. obtained it does not appear, probably never. Looking at the figures in another way, we find that at the end of six years less than half of the number of students had obtained a qualification for registration and at the end of seven years only two-thirds. Those who took eight years constituted 13 per cent. of the total, or almost as large a proportion as those who qualified in the minimum period of five years.

The statistics in the three kingdoms, it is true, show considerable variations, 23 per cent. of the students in Scotland and 12 1-2 per cent. of the students in Ireland obtaining a qualification in the minimum period, whereas in England only a little over 7 per cent. were thus successful. Looking at the matter broadly, however, we shall not be far wrong in asserting that no student entering a medical school can expect to leave it with a qualification in less than six years, and that neither he nor his parents need be surprised if the period extends to seven years.

Aspirants to the ranks of the profession must give proof, before they are eligible to registration as medical students, of an "efficient knowledge" of at least the following subjects: English in its various branches, including history and geography, Latin, mathematics, including arithmetic, algebra and geometry, and either Greek or a modern language, and that they are at least 16 years of age.

It is only from the date which appears against his name in the *Students' Register* that the medical student's career officially begins; thereafter five years must pass before he can present himself for final examination for any diploma which entitles its lawful posessor to registration as a qualified medical practitioner under the Medical Acts.

An academic year is divided into a winter and a summer session. The minimum 5 year period must be one of bona fide study, and during its course education in the following subjects must be pursued and examinations passed:

Physics, chemistry, biology, anatomy, physiology, materia medica and pharmacy, pathology, therapeutics, medicine, including medical anatomy and clinical medicine, surgery including medical anatomy and clinical surgery, midwifery (25 cases must be attended), gynecology, including diseases peculiar to women and new-born children, vaccination, forensic medicine, hygiene, mental diseases.

The regulations also generally necessitate definite study for stated periods of diseases of children, of the larynx, ear and nose, of the skin, of ophthalmology.

"A student to obtain a registrable qualification in the minimum period of five years, or fifty-seven months, must have a certain amount of good luck; in other words, he must keep in good health through every term, and never fail at a single examination."

One of the most striking differences between British and American regulations is observable when the question of degrees is discussed. In the United States, but not in Canada, it is, we believe, the universal custom to grant the doctorate to the student who has successfully passed his college examinations. In the British islands this is never done. These medical schools may be divided into universities and colleges, only the former granting the doctorate, and that to those alone who have undergone a farther period of probation, and passed some form of examination, at least one year after the Bachelor's degree has been taken at the termination of the ordinary student's career of 5 years or more. The colleges, such as the Royal Colleges of England and of Edinburgh, give memberships or licenses as evidences of the ability of their owners to practice medicine. These are ample for the purpose, and probably the vast majority of British practiioners possess no other diploma. But such as desire to show evidence of more prolonged application proceed to take one or more of the higher diplomas.

Licentiates and members, as such, may proceed to take the fellowship of a college, a degree which is always convincing evidence, to those who know its value, of both ability and hard work. Bachelors have the advantage of being able to take not only the doctorate of their university, but also the membership and afterwards the fellowship of any of the colleges by merely passing their examinations without attending their classes. But the fellowship of the Royal College of Physicians of London is a purely honorary degree. Besides strictly medical diplomas there are others which have an important bearing on medicine, as for instance the Bachelor and Doctorate of Science in Public Health, or the Diploma in Public Health, one of which is expected of applicants for public health appointments, and each of which is obtainable only after a considerable period of study in the theory and practice of subjects relating to Preventive Medicine.

---

### TO IMPROVE MEDICAL EDUCATION AND MEDICAL PRACTICE LAWS.

---

At a call meeting of the Regular Board of Medical Examiners on June 10, 1908, at Macon, Ga., there were present Drs. E. R. Anthony, J. B. S. Holmes, F. D. Patterson of the Board, Dr. G. H. Noble of Atlanta College of Physicians and Surgeons. The following questions were discussed and agreed upon:

First.—To increase the medical course to four years of at least six months each in four separate and distinct years.

Second.—That medical students in attendance upon medical colleges be allowed t stand examination at the end of the second year in histology, bacteriology, materia medica, chemistry and physiology before the Regular State Medical Examining Board if they have successfully passed these branches in the colleges they are attending, and this examination to be final if the required percentage of the Regular State Medical Examination

Board is made. If they fail to make the required percentage, they be allowed another examination after their graduation. A certificate from the dean of the college must be presented by each applicant.

Third.—That the Secretary of the State Regular Board of Medical Examiners be authorized to grant temporary license after satisfactory examinations by him.

Fourth.—To amend the law to authorize the State Regular Board of Medical Examiners to revoke licenses for cause.

Fifth.—The Regular Board of Medical Examiners be authorized to accept only applicants for examination who are graduates from colleges belonging to the American Medical College Association and the Southern Medical College Association, or other medical colleges that are recognized by the regular or mixed State Licensing Boards of their respective states.

Sixth.—That term of office of the members of the State Regular Board of Medical Examiners shall be four years instead of three.

Seventh.—Vacancies shall be filled on the State Regular Board of Medical Examiners as follows: Members of this Board, the President of the State Association and the Chairman of the Counsel shall present the names of three worthy and well qualified physicians for each vacancy to the State Association for their ratification, and from these names the Governor shall make the appointments.

Eighth.—That State Regular Board of Medical Examiners recommend to the colleges the preliminary educational requirements of a high school diploma or a second-grade teachers license furnished by the County School Commissioner.

E. R. ANTHONY, Sec'y.

J. B. S. HOLMES, Chairman.

---

### SOUTHERN MEDICAL ASSOCIATION.

Again we would like to call attention to the next annual meeting of the Southern Medical Association which will be held in Atlanta November 10, 11, and 12, 1908. This meeting promises to be of much interest as it probably will have a larger attendance than has had any southern meeting of the medical men heretofore.

Many excellent papers will be read and will demonstrate the careful scientific work being done in the South. No physician who can possibly come can afford to miss this meeting. Short papers with a point and lively discussions are earnestly desired.

Southern Passenger Association has authorized a rate of one and one-third fare, plus not exceeding 25 cents, from all points embracing in the Southern Medical Association east of the Mississippi river. (Rate west of the river will no doubt be the same.—Secretary.) Date of sale November 7, 8, 9, good until November 14, leaving Atlanta, with three days transit limit.

The following letter has been sent to Georgia physicians who are urged to attend this meeting:

Dear Doctor:

The Southern Medical and Surgical Association will meet in Atlanta, November 10th, 11th, and 12th. As you probably know this association has already attained what it has intended to be, an association for the advancement of science and particularly to bring together the workers in the South.

The American Medical Association is so big and usually meets so far away from home that it does not furnish a medium for the exchange of ideas among the southern physicians and surgeons.

There is a great deal of splendid work being done in the South which does not receive a large southern audience. This association, then, will be the means of affording its members the opportunity to see and hear good work that will be a stimulus to better work, and what is not least, will furnish pleasant association with our fellows of the surrounding states once a year.

It is particularly desirable that Georgia should be well represented in the coming meeting in Atlanta. Every physician who is a member of the State Association is eligible to membership in this Association, and upon payment of the dues becomes a member. I wish, therefore to extend to you, in behalf of the Association, a most cordial invitation to be present at the meeting in Atlanta in November.

The members of the profession of Atlanta and looking forward with the greatest pleasure to this meeting, and shall be delighted to welcome you.

If you desire to join, kindly fill out the enclosed blank and make out a check for two dollars, payable to Doctor Oscar Dowling, Secretary, and send them both to me. I will forward the check to Dr. Dowling.

Very respectfully yours,

MICHAEL HOKE, M. D.

---

### COLLECTION DEPARTMENT OF THE FULTON COUNTY MEDICAL SOCIETY.

Believing that the best interests of the medical profession could be promoted by a systematic effort to collect accounts from patients who were well able to pay the physician, but *would not*, the Fulton County Medical Society appointed a committee to consider the matter of arranging for such a collection. After much deliberation and many conferences with various prospective collectors, the committee finally settled upon the definite plan which is briefly stated in the following letter which has been sent to each member of the society, and explains itself.

Dear Sir: I beg to inform you that the "Collecting Committee" of the Fulton County Medical Society has made an arrangement with Mess. Napier, Wright & Cox, Attorneys, by which these gentlemen have agreed to handle the collections of our association on the following terms. Claims of $5.00 and less, 50 per cent; $5.00 to $10.00, 25 per cent; $10.00 to $20.00, 20 per cent.; $20.00 to $50.00, 12 per cent; and all claims amounting to over $50.00, 8 per cent.

They have agreed to render statements on the first of each month to each doctor who places his claims in their hands, which statements shall make known the condition of the claims in their hands, such record to be the property of the secretary of the association.

They will also make up for the members who send in accounts, a list giving such information as may be desired.

Trusting that this may be of value to you, I am,

Very truly yours,

J. ROSS SIMPSON,

Secretary Fulton County Medical Society.

The daily press soon secured the facts concerning the above plan of collecting bad accounts and gave considerable space to an exaggerated and hysterical description of the "dead beat list," "the doctors boycott," etc. The Atlanta Constitution finally dealt with the matter in a very sensible editorial which brought to a close the sensational features of the plan of the collection committee. The standard of the medical profession undoubtedly can be raised by making reasonable and systematic efforts to collect their accounts. To keep abreast the times, to buy new books, new instruments, office equipment and suitable conveyances, and to attend the various medical associations and post-graduate schools require not *much more* than *could* be collected, by the average physician, *above* his usual income. That better equipment and training will increase the physicians efficiency no one will gainsay, consequently the patient will not be a loser, but a gainer, by reasonably prompt payment, even though some effort on the part of the collector is required in making the settlement.

All physicians of Atlanta are urged to avail themselves of the reasonable rates of the collectors and the other advantages that will accrue from this plan.

---

### "MEDICAL CONCENSUS" SUSPENDS PUBLICATION. THE JOURNAL-RECORD BUYS SUBSCRIPTION LIST.

---

*The Medical Concensus* has ceased publication, and hereafter the JOURNAL-RECORD OF MEDICINE will be sent to their subscribers, thereby carrying out the integrity of the subscription list of the *Medical Concensus*. We trust this arrangement will prove satisfactory to the friends of both journals.

### MILITARY SURGEON'S CONVENTION.

The seventeenth annual meeting of the Military Surgeons was held in Atlanta, October 13, 14, 15 and 16, in the assembly rooms of the Piedmont Hotel, with the following officers officiating:

President, Assistant Surgeon General George Tully Vaughan, P. H. M. H. S.; first vice-president, Rear Admiral Presley D. Rixley, U. S. N.; second vice-president, Colonel Joseph K. Weaver, N. G. Pa.; third vice-president, Colonel William C. Gorgas, U. S. A.; secretary, Major James Evelyn Pilcher, U. S. V.; treasurer, Major Herbert A. Arnold, N. G., Pa.; assistant secretary, Captain J. Carlisle DeVries, N. G. N. Y.

In addition to the scientific meetings, at which many excellent papers were read on subjects of particular interest to military surgeons, there were public meetings at the State Capitol, a reception and ball at the Piedmont Driving Club, an automobile ride and a barbecue at the Atlanta Cue Club.

The Atlanta physicians felt highly honored to be able to entertain so many distinguished military surgeons and extended to them a hearty welcome.

Among the delegates enrolled upon the registration book were:

Colonel George Tulley Vaughan, Major James E. Pilcher, W. H. Marsh, Maryland; Colonel James K. Weaver, Pennsylvania; Major H. A. Arnold, Pennsylvania; Captain J. Carlisle DeVries, New York; Captain John Vernon Frazier, Michigan; Brigadier General F. Elbert Davis, New York; Colonel Alejanois Ross, Mexico; Lieutenant Colonel Jose Barbosa Leao, Portugal; Colonel George Brown, Georgia; Medical Director John C. Wise, Washington, D. C.; Lieutenant Colonel W. H. W. Elliott, Indian medical service; Major J. W. Duncan, Georgia; Captain C. B. Walls, Illinois; Lieutenant Colonel M. W. H. Russell, R. A. M. C. England; Major E. S. Bet, Canada; Brigadier General Charles C. Foster, Massachusetts; Lieutenant Colonel G. P. Marquis, Illinois; Major G. H. Halberstadt, Pennsylvania; Major James R. Nankeville, Indiana; Major T. W. Evans, Wisconsin; Captain George H. Hidershide, Wisconsin; Major Dan S. Burr, New

York; Major T. W. Halbert, Tennessee; Captain G. Morgan Muren, New Jersey; Major Henry Aaron Jones, Rhode Island; Colonel Joachein B. Weintraub, Illinois; Lieutenant Montifiax W. Houghton, Rhode Island; Gustavus M. Blech, Illinois; B. J. Weitinsprous, North Carolina; Brigadier Alexander I. Stone, Maine; Colonel John S. Edwards, Wisconsin; Surgeon W. T. Thackery, Illinois; Captain P. I. Butler, Massachusetts; Major Henry Allers, New Jersey; Lieutenant W. D. Lyman, Michigan; Major Thomas J. Sullivan, Illinois; P. M. Garrington, Surgeon U. S. P. H. and M. H. S., New Mexico; Major Charles B. Ewing, Georgia; Captain H. H. Tuttle, Illinois; Major Frank H. Holmes, North Carolina; Lieutenant Thomas F. Duhigg, Iowa; Major Erwin M. Fuller, Maine; Major J. W. Heany, Cuba; Major E. L. Martindale, Iowa; Captain B. F. Bradbury, Maine; Colonel J. B. O'Neill, Maine; Major J. F. Lynch, Virginia; Captain A. P. Kalliontzio, Illinois; Colonel F. S. Nicholson, Nebraska; M. H. Simons, medical United States navy, Pennsylvania; Major C. Lynch, Wisconsin; Captain S. H. Green, Georgia; Surgeon C. P. Wertenbaker, Virginia; Lieutenant Arthur R. Jarrett, New York; Major J. C. Minor, Arkansas; Lieutenant Colonel A. S. Stayer, Pennsylvania; Medical Director M. J. White, P. H. and M. H. S., Michigan; Colonel Valery Harvard, Washington, D. C.; Major George S. Crampton, Pennsylvania.

